# Advancing sustainable financing through pandemic bond: a Bayesian model averaging approach to liquidity premia

**DOI:** 10.1186/s40854-026-00991-0

**Published:** 2026-11-06

**Authors:** Tianen Yao, Xinyu Yang, Yuhang Pan, Zhihan Cui, Ming Xu

**Affiliations:** 1 Department of Global Health, School of Public Health, Peking University, No.38, Xueyuan Road, Haidian District, Beijing, 100191 China; 2 Institute for Global Health and Development, Peking University, Beijing, 100871 China

**Keywords:** Pandemic bond, Liquidity premium, ESG, Insurance cycle, Health sustainability

## Abstract

Pandemic bonds, as a subtype of catastrophe (CAT) bonds, are designed to transfer public health risks to capital markets, yet they face substantial liquidity risk due to their nascent market structure and limited risk design. Using a relatively small sample from 2020 to 2024, we apply Bayesian model averaging (BMA) and interpretable machine learning (IML) to examine the associations of macroeconomic and bond-specific characteristics with liquidity premia across market phases. We find that the average liquidity premium of natural catastrophe bonds (1364 bps) is substantially higher than that of pandemic bonds (452 bps), potentially reflecting differences in bond characteristics and contractual structures. The estimates suggest that higher ESG scores and higher disclosed health scores are associated with lower estimated pandemic-bond premia, with health-score categories receiving slightly greater posterior inclusion support than ESG in the joint specification. Pandemic bond premia are higher during hard market phases, while several predictors receive greater posterior inclusion support during soft markets. Overall, the results suggest that incorporating a health-specific index, alongside insights from traditional CAT and green bond design, could enhance the resilience of pandemic bonds and strengthen their role in sustainable development.

**Supplementary Information:**

The online version contains supplementary material available at https://doi.org/10.1186/s40854-026-00991-0.

## Introduction

Climate change-induced social inequality seems to have been masked since the COVID-19 outbreak, but it persists and is gradually worsening (Smiley et al. 2022; Vandentorren et al. 2022). Many vulnerable populations lack access to adequate insurance coverage or healthcare following catastrophes (Schwarz et al. 2021; Carleton and Hsiang 2016), particularly during the postpandemic era (Shi et al. 2022). Given that the pandemic has caused an estimated USD 1 trillion in losses, and natural catastrophes have led to USD 202.7 billion in losses in 2023, there remains a vast gap in government and private economic aid (EM-DAT 2024; Quantin and Tubert-Bitter 2022; Kotz et al. 2024). In response to these challenges, sustainable finance, broadly defined as financial investments that are devoted to social equality, has received extensive attention (Lo and Zhang 2024). It can shift climate risk to capital markets (van Benthem et al. 2022). Nevertheless, financing tools in the health sector, which mainly rely on development assistance, are relatively scarce (World Health Organization (WHO) 2022). It is urgently needed to develop more improved financing tools to address the challenge in public health.

Traditional green financial tools comprise green bonds and ESG investments (Lo and Zhang 2024). Notably, catastrophe (CAT) bonds are a popular tool in the insurance-linked securities (ILS) market as a means for businesses and institutions to mitigate the financial risks of extreme climate events (Zhao and Yu 2020). For instance, disadvantaged groups have access to insurance payments (Fig. S1), facilitated by CAT bonds (Jardine et al. 2020). Similarly, the Fishery Mutual Insurance (FMI), a government-supported not-for-profit mutual insurance program in China, covers production costs, helping fishers remain operational despite significant losses (Jardine et al. 2020). More recently, companies have begun linking CAT bond proceeds directly to sustainability goals. In March 2024, Tokio Marine Holdings, Inc. was the first firm to invest the proceeds of its CAT bond issue in sustainable development, signaling a new direction for corporate engagement in environmental risk financing (Artemis Deal Directory 2024).

At present, research on the liquidity risk of pandemic bonds remains relatively scarce, particularly lacking studies on its macroeconomic cycle. First, we examine the liquidity risk factors for pandemic bonds by categorizing them as CAT bonds. CAT bonds are characterized by high excess returns and premia, albeit with low liquidity (Götze et al. 2020). For example, the average premia reached 717.37 basis points (bps) from 2002 to 2016 (Brim and Wenham 2019). Prior research has typically attributed the risk of high liquidity premia to macroeconomic and bond-specific indices (Götze and Gürtler 2020). For bond-specific variables, the risk of default is highly publicized, and therefore, credit ratings are critical (Schwert 2017). Compensation-triggered CAT bonds are likely to have higher risk premia than noncompensation-triggered CAT bonds (Dieckmann 2019). The most commonly used index reflecting the loss distribution is expected loss (EL), which is positively associated with bond premia (Herrmann and Hibbeln 2021). The higher the number of hazard locations and types of issuances, the higher the premium is likely to be (Hagendorff et al. 2013; Lane 2022). In addition, developed countries play a leading role in bond issuance since the weighted average cost of capital of low- and middle-income countries is higher (Ameli et al. 2021). Regarding macroeconomic factors, financial markets are usually cyclical (Götze and Gürtler 2020). Premia is lower during “soft” markets and higher during “hard” markets (Lane 2022). In addition, the S&P (Standard & Poor’s) 500 index, Corporate Credit Spread (Corp. Spread), and term structure are determinants (Schwert 2017; Herrmann and Hibbeln 2021).

Moreover, the impact of ESG investments on the liquidity risk of pandemic bonds is examined. ESG scores have a wide range of applications in impact investing. Despite discrepancies in scoring and susceptibility to higher market risk, ESG scores can help companies achieve their financial goals and enhance their social value (Avramov et al. 2022). For green bonds, their issuance may significantly support the financing of the construction (and/or renovation) of sustainable greenfield (and/or brownfield) infrastructure. However, some scholars have found that the more investors contribute to realizing such infrastructure assets for ESG investments, the higher the rate of return that investors are willing to forgo (Baldi and Pandimiglio 2022). Therefore, we are also curious to see how ESG scores contribute to pandemic bonds regarding health financing and whether we can provide a more refined ESG scoring system applicable to the health component.

In summary, research on the liquidity premia of pandemic bonds is limited. Beyond their role as insurance-linked securities, pandemic bonds are increasingly framed within ESG-oriented investment strategies, reflecting their potential to support health financing through risk transfer and sustainability considerations. This study contributes to the literature on pandemic bonds by providing evidence from a relatively underexplored health-financing context. The aim of this study is to (1) reveal the global trend of pandemic bonds in the context of global health; (2) quantify the contribution of macroeconomic and bond-specific factors to the liquidity premium of pandemic bonds while comparing it with ILSs such as CAT bonds; (3) assess the role of sustainability signals, including ESG indicators and a health-specific index, in the pricing of pandemic bonds relative to green bonds; and (4) compare the heterogeneity of the different market stages.

## Literature review

### Overview of bond financing for health

Pandemic bonds, as a subset of CAT bonds, have emerged as critical innovative financial instruments in pandemic preparedness and response. Initiated by the World Bank’s Pandemic Emergency Financing Facility (PEF) following the 2014–2016 Ebola crisis, their core function is to trigger rapid funding for pandemic mitigation when predefined thresholds are met (Brim and Wenham 2019). The principal is held by a special purpose vehicle (SPV) in the form of highly rated collateral, which is paid to the hedging (re)insurer to cover its losses if trigger conditions specified in the bond indenture are met (Fig. S2). However, pandemic bonds rely heavily on low-probability events, which conflicts with the investment philosophy of anticipation based on probability distributions (Giuricich and Burnecki 2019). In practice, further limitations persist: PEF’s stringent trigger mechanisms and prioritization of private sector interests have undermined its alignment with global health security, leaving gaps in equitable access to financing during crises (Zheng and Mamon 2023).

As a representative of emerging economies, China took the lead in issuing pandemic-related bonds during the COVID-19 pandemic, providing a localized solution for public health financing and alleviating structural funding shortages (Shi et al. 2022). Between 2020 and 2021, China issued over 2.3 trillion yuan in pandemic bonds. Guided by Ministry of Finance policies, these bonds were issued through multiple channels, including local government special-purpose platforms, policy banks, and enterprises. Together, these issuances injected critical liquidity into medical infrastructure development, vaccine logistics, and the resumption of corporate operations. They demonstrated significant effectiveness, not only boosting market sentiment but also yielding positive cumulative abnormal returns for issuing companies. Their credit spreads showed a strong correlation with regional pandemic severity, with particularly pronounced effects in hard-hit provinces such as Hubei (Shi et al. 2022). Unlike models relying on international donations, such as the World Bank's PEF, China's approach centers on national policy coordination and localized implementation, highlighting the advantages of domestic adaptation (Wei and Han 2021). However, this approach presents both achievements and limitations. While it has stabilized local markets and enhanced short-term financing capacity, questions remain regarding its long-term efficiency, data transparency, and replicability in resource-constrained regions. These cases highlight two urgent research needs: first, identifying the drivers and barriers to pandemic bond effectiveness, notably in resource-constrained settings; second, addressing methodological challenges such as limited data availability.

Relevant innovations in global health financing, such as vaccine bonds issued by the International Finance Facility for Immunization (IFFIm), provide valuable parallels. Since 2006, IFFIm has mobilized over $8 billion to support vaccine access, demonstrating how structured financial instruments can scale health interventions (IFFIm 2022). However, pandemic bonds differ fundamentally from vaccine bonds: while the latter target predictable, long-term health needs, pandemic bonds must navigate high uncertainty in crisis timing and severity, making their impact assessment far more complex. This distinction underscores the need for tailored analytical frameworks to evaluate how pandemic bonds can better align with dynamic public health demands.

In this study, we use the term “pandemic bonds” to refer to pandemic-related CAT bonds in our Bloomberg sample, including both internationally issued instruments (e.g., PEF-type structures) and China’s COVID-19 response issuances. Vaccine bonds are discussed only as a related benchmark in global health financing and are not included in the empirical sample.

### Macroeconomic cyclicality and liquidity risk

Economic cycles influence bond markets through two key channels: default risk and risk appetite (Gilchrist and Zakrajšek 2012). During economic expansions, robust corporate profitability and rising asset values reduce default risk, narrowing the expected default component of credit spreads. In addition, investors exhibit low risk aversion and greater willingness to bear credit risk, leading to relatively active traditional bond trading and higher liquidity. In economic downturns, elevated corporate default risk and heightened investor risk aversion lead investors to demand higher risk premiums, increasing transaction costs and reducing liquidity in bond markets. In insurance-linked securities markets, macroeconomic conditions interact with insurance market cycles, commonly described as hard and soft phases. Such stylized market classifications are widely adopted in the insurance and reinsurance literature (Tesselaar et al. 2020; Götze and Gürtler 2020). For CAT bonds, soft markets are associated with lower reinsurance costs and higher shareholder value, as issuers lock in favorable pricing rather than primarily hedging risk (Hagendorff et al. 2013). Pricing mechanisms also differ across market phases (Götze and Gürtler 2020). In hard markets, where premia are relatively high, underwriter experience and reputation play a stronger mitigating role, while in soft markets, abundant capital compresses spreads and weakens the relevance of underwriter characteristics.

The liquidity premium in CAT and pandemic bonds has been measured through several approaches. Some studies employ bid-ask spreads as a direct, market-based proxy reflecting trading costs and market depth (Herrmann and Hibbeln, 2023). Others use structural variables such as issue volume and remaining maturity to approximate relative liquidity (Gürtler et al. 2016). A more general and robust approach adopts the yield-to-maturity (YTM) minus LIBOR differential, which captures investors’ required compensation for both funding and liquidity risks when transaction-level data are unavailable (Braun 2016). Given the limited secondary market activity in CAT and pandemic bonds, the YTM-LIBOR method provides a consistent and comparable measure of liquidity premium across bonds and time.

### ESG

Green bonds have become a cornerstone of sustainable finance, playing a crucial role in funding projects with positive environmental outcomes (Bachelet et al. 2019). Their introduction has brought significant attention to ESG principles, which now heavily influence modern investment decisions (Zerbib 2019). However, the field faces key challenges. There is a lack of agreement among rating providers, which leads to confusing data since corporate virtue is often in the eye of the beholder (Berg et al. 2022; Christensen et al. 2022). This ambiguity creates a risk of "greenwashing", where labels may overstate an asset's true impact and undermine investor trust (Bachelet et al. 2019). While ESG rating disagreement is a recognized factor affecting stock returns, stocks with strong environmental and social credentials have shown impressive resilience during market crises (Gibson et al. 2021; Albuquerque et al. 2020). The lessons learned are critical: green bond certification can create a small price premium but may harm tradability under stress, often because a niche group of mission-driven investors narrows the pool of active buyers (Ehlers et al. 2020). This segmentation can concentrate ownership, which in turn drives up resale costs and widens bid-ask spreads when large investors trade (Baker et al. 2018).

The insights from green bonds and similar instruments provide a valuable framework for understanding pandemic bonds. Our research focuses on how these ESG investment dynamics are associated with their liquidity risk. It is believed that pandemic bonds should serve a greater social purpose, especially in structuring finance for high-impact healthcare projects (Rizzi et al. 2018). This aligns with findings that strong social capital enhances firm value during a crisis and that corporate immunity was a key factor in performance during the COVID-19 pandemic (Lins et al. 2017; Ding et al. 2021). The core hurdle, however, is that current broad ESG metrics often fail to capture health-specific dimensions relevant to pandemic risk, offering insufficient guidance to the investor community (WHO 2022; International Capital Market Association (ICMA) 2023). Therefore, we are also curious to see how ESG scores contribute to pandemic bonds regarding health financing and whether we can provide a more refined ESG scoring system applicable to the health component.

To better balance a bond's social impact with its market tradability, labeling frameworks need to move beyond generic ESG classifications and incorporate health-specific criteria, including preparedness-related commitments, clarity in use of proceeds, and transparency in governance and reporting (WHO 2022; ICMA 2023). Furthermore, the market's infrastructure requires an upgrade through stronger third-party verification and more consistent reporting standards to combat issues such as greenwashing (Ehlers et al. 2020). Existing studies also show that deploying data analytics, such as feature selection and clustering methods, can help create more transparent and relevant metrics for assessing financial risk (Kou et al. 2014, 2021). By taking these steps together, it is possible to reduce ESG classification risk, attract a broader base of investors, and ultimately mitigate liquidity risk.

In summary, this literature review builds on three dimensions of existing research. First, studies on pandemic bonds have examined their role in health crisis financing, yet most remain descriptive and overlook how market liquidity and regional heterogeneity shape their effectiveness. Second, research on macrofinancial cycles shows that bond markets are strongly influenced by changes in default risk, investor sentiment, and liquidity across different economic phases. These cyclical dynamics affect both corporate and CAT bond pricing and highlight the need to measure liquidity premia consistently over time. Third, evidence from ESG-related studies highlights how sustainability signals influence pricing and investor behavior, but current frameworks lack health-specific standards. As summarized in Table S1, the findings underscore the importance of developing holistic frameworks that quantify liquidity premia across macrofinancial cycles and incorporate ESG reports, contributing to the development of more resilient and transparent pandemic bond markets.

## Data and methods

### Data

#### Sample selection

We mainly collect the data, including maturity, volume, and yield, from Bloomberg (Bloomberg 2024). The data on the bonds’ ELs, issue volumes, trigger mechanisms, insured peril types, and regions are taken from the Artemis Deal Directory (Artemis Deal Directory 2024). Treasury rates are obtained from the Federal Reserve Bank (Federal Reserve Bank 2024), and the LIBOR (London Interbank Offered Rate) is from the Market Watch (Market Watch 2024). We also collect the S&P 500 index from Thomson Reuters (Thomson Reuters 2024). The sample selection procedure in our study is as follows: (1) the range of time is from 2020 to April 2024, and there are 719 pandemic bonds and 429 natural CAT bonds, (2) some bonds contain substantial missing values, thus being deleted, and (3) some indicators had more than 40 percent missing data and are therefore deleted. Finally, 290 pandemic bonds and 360 natural CAT bonds are included in our study.

#### Variables

##### Macroeconomic factors

Macroeconomic factors are employed to control for overall market development, including the S&P 500, Corp. Spread, and term structure. The Corp. Spread refers to the credit spread of US corporate bonds across different rating classes and maturities between one and three years (Schwert 2017). In addition, we construct the “term structure” variable, defined as the difference between the ten-year U.S. treasury rate and the three-month U.S. treasury rate (Herrmann and Hibbeln 2021).

##### Bond-specific variables

We include a series of bond-specific control variables to avoid omitting important determinants of CAT bond premia in subsequent analyses. In addition, we create the log(Volume) variable, which re-expresses the natural logarithm of bond issuance as a potential proxy variable for bond liquidity. We capture the association between bond maturity and premia by introducing a maturity variable. Finally, we set a series of dummy variables for different risk types, risk regions, and bond rating categories, all of which are defined in Table 1.

**Table 1:** Summary statistics of natural CAT bonds and pandemic bonds

Natural CAT bond						Pandemic bond				
Variables	Obs	Sd	Min	Mean	Max	Obs	Sd	Min	Mean	Max
Premia	216	7.62	5.73	13.64	78.19	187	6.44	0.25	4.52	77.44
log(Volume)	351	0.68	9.90	13.97	15.56	284	1.79	9.89	15.02	20.03
YTM	216	7.61	5.79	13.67	78.20	187	6.44	0.25	4.53	77.45
Coupon	356	5.88	0	10.91	28.38	289	2.37	0	2.34	12.45
Rating	337	0.64	0	0.14	5	174	1.90	0	3.68	6
Maturity	360	1.22	0.48	3.55	12.18	285	9.57	0.12	7.89	100.72
Trigger	338	0.05	0	0.997	1					
Expected loss	313	2.07	0.001	2.305	19.08					
Peril types										
Earthquake	338	0.50	0	0.53	1					
Hurricane	338	0.35	0	0.14	1					
Wind	338	0.48	0	0.64	1					
Wildfire	338	0.39	0	0.19	1					
Other	338	0.46	0	0.29	1					
Peril location										
Europe	338	0.29	0	0.092	1					
North America	338	0.40	0	0.80	1					
Asia/Australia	338	0.28	0	0.083	1					
Other	338	0.23	0	0.056	1					
Macro										
S&P 500	360	570.03	2585	4252	5254	290	572.05	2585	3303	5254
Term structure	360	1.30	-1.57	0.21	1.99	290	0.48	-1.57	0.67	1.99
Corp	360	0.20	0.40	0.69	1.46	290	0.32	0.40	0.97	1.46
Pandemic bond										
Type						290	0.46	0	0.30	1
Issue market						290	0.50	0	0.50	1
ESG score						95	15.42	10.99	39.24	71.29
Health score						290	3.30	0	4.22	10

(1) Only one natural CAT bond does not trigger indemnity. (2) The Artemis Deal Directory recorded one pandemic bond, which was the first pandemic bond issued by the World Bank in 2017. (3) Wind bonds are in terms of wind, windstorm, and storm. (4) Other peril locations include South America and the globe. (5) Other peril types cover volcanic eruption, meteorite impact, hail, flood, cyber risk, terrorism risk, all lines of business, financial guarantee risks, medical benefit claims levels, tsunami, landslide, hydrothermal, etc. (6) Peru once issued a pandemic bond with an extraordinarily long maturity of up to 100 years in 2021. (7) The ‘Obs’ indicates observations

The average rating is assessed based on the S&P rating obtained for the CAT bond issued (or based on Moody's and other ratings if no S&P rating is available). To make ratings comparable across agencies, we map the original rating labels into a unified ordinal scale: 0 (No Rating), 1 (S&P B or Moody’s B), 2 (S&P BB or Moody’s Ba), 3 (S&P BBB or Moody’s Baa), 4 (S&P A or Moody’s A), 5 (S&P AA or Moody’s Aa), and 6 (S&P AAA or Moody’s Aaa). For pandemic bonds, “Type” takes the value of 1 if the issuer of the bond is government and 0 (corporate) otherwise. “Issue market” takes the value of 0 if the issue market is the domestic market and 1 otherwise. To capture health-related sustainability characteristics that are not reflected in conventional ESG metrics, we construct an issuer-level health score based on publicly available disclosures (Table S2). The score aggregates several health-specific indicators related to pandemic preparedness, health system investment, use-of-proceeds alignment, and reporting transparency (ICMA 2025; WHO 2020). Each indicator is coded using a simple disclosure-based scheme, and the resulting score is grouped into low, medium, and high categories for empirical analysis.

EL represents the average loss an investor expects to incur over a given period divided by the amount of capital invested (Gürtler et al. 2016). It is typically calculated as EL=PFL×CEL, where PFL represents the probability of the first loss and CEL represents the conditional EL. Investors can use EL multiplied by the coupon rate paid to assess the attractiveness of an insurance facility relative to other comparable transactions. However, EL metrics are limited by their lack of timeliness and potential disconnect from market expectations.

#### Descriptive statistics

Compared with pandemic bonds from Bloomberg, the Artemis Deal Directory provides additional natural CAT bond indices, including peril location, peril type, trigger compensation, and EL. ESG reports and scores are unavailable for the natural CAT bond sample. In our analytical sample, pandemic bonds have lower average coupon rates (2.34% versus 10.91%), higher mean log issue volume (15.02 versus 13.97), and longer average maturity (7.89 versus 3.55 years) than natural CAT bonds (Table 1). Rating profiles also differ: most natural CAT bonds in the sample, including all hurricane bonds, are unrated, whereas pandemic bonds with available ratings generally occupy higher rating categories.

### Methods

#### Liquidity premia regression

Following Gürtler et al. (2016), we proxy the liquidity premium using the yield spread over LIBOR for each bond *i*. ${YTM}_{i}$ denotes the YTM of bond *i*, measured using Bloomberg secondary-market mid-yield-to-maturity observations, representing the annualized return an investor would receive by holding the bond until maturity, reflecting the bond's overall return level; ${LIBOR}_{t}$ is matched to the bond's remaining maturity (e.g., a 1-year bond corresponds to 1-year LIBOR, a 3-year bond to 3-year LIBOR), serving as the risk-free rate benchmark (Gürtler et al. 2016). After removing the risk-free return,${Premia}_{i}$ represents the excess yield demanded by investors, reflecting a composite risk premium with an important liquidity-related component, rather than a pure liquidity effect.

1 $$\begin{array}{c}{Premia}_{i}={YTM}_{i}-{LIBOR}_{t}\end{array}$$

Throughout the manuscript, we follow the CAT bond literature and refer to this spread as the liquidity premium while interpreting it as a proxy rather than a pure liquidity effect. It should be noted that the traditional USD LIBOR quotations were officially discontinued after June 2023. Data from subsequent periods up to April 2024 are synthetic LIBOR rates mandatorily published by the UK FCA (Financial Conduct Authority), which are constructed based on SOFR (Secured Overnight Financing Rate) plus a fixed credit spread. Such synthetic LIBOR is intended only for the transition of legacy contracts and does not represent actual market transaction rates.

Then, we construct a normal linear regression to examine the associations between risk factors and premia.

2 $$\begin{array}{c}{Premia}_{i}=\alpha +{\beta}_{i}{X}_{i}+{\gamma}_{i}\end{array}$$

where α denotes the constant term, CAT bond *i* = 1, 2, …, n, ${X}_{i}={({x}_{i1},\dots , {x}_{ip})}{\prime}$ denotes a vector of explanatory variables with the coefficient vector ${\beta}_{i}={({\beta}_{1},\dots , {\beta}_{p})}{\prime}$, and ${\gamma}_{i}$ indicates a random error.

#### Bayesian model averaging

Model uncertainty is particularly relevant in our setting given the relatively small sample sizes of pandemic and CAT bonds. Rather than relying on a single selected model, Bayesian model averaging (BMA) explicitly incorporates model uncertainty by averaging across all candidate models weighted by their posterior probabilities (Wasserman 2000; Hinne et al. 2020). BMA is crucial in limited-data contexts because it provides more stable inference by reducing sensitivity to any single model choice.

A key output of BMA is the posterior inclusion probability (PIP), which measures how strongly the data support including a given regressor across competing models. For a regressor Δ, the posterior inference is averaged across all models ${M}_{j}$ in the model space (Hoeting et al. 1999):

3 $${P}_{\Delta |y}={\sum}_{j=1}^{{2}^{k}}{P}_{\Delta |y, {M}_{j}}P\left({M}_{j}|y\right)$$

where k is the number of candidate regressors and $P({M}_{j}\mid y)$ denotes the posterior probability of model ${M}_{j}$.

To avoid imposing strong prior beliefs, we adopt a weakly informative prior specification commonly used in the BMA literature. Specifically, we employ Zellner’s g-prior for regression coefficients and set the variance-scaling parameter g following Fernandez et al. (2001). We further assume a uniform prior over the model space, treating all candidate models as equally likely a priori. BMA addresses model uncertainty rather than endogeneity, and the estimates are interpreted as conditional associations.

#### Hard, soft, and neutral markets

Prices in insurance and reinsurance markets follow a cyclical pattern, alternating between hard markets with tighter pricing and higher premia and soft markets characterized by more favorable pricing and lower premia. It is therefore necessary to control for these cyclical effects in the empirical model to investigate the drivers of premia in different market phases. To identify this price volatility in CAT bonds and to distinguish it from the effects of broader capital market conditions (e.g., macroeconomic developments or price levels in fixed-income markets), we make further modifications to the price index. Specifically, we standardize the monthly price index (PI) through a simple regression on the relevant macroeconomic variables (corporate spreads, S&P 500 index, term structure and 1-year Treasury rates), which can be expressed as (Götze et al. 2020):

4 $$\begin{array}{c}{PI}_{i}=\delta +{\theta}_{i}{Z}_{i}+{\mu}_{i}\end{array}$$

where *PI* is the volume-weighted market-to-market price of CAT bonds in the secondary market, δ denotes the constant term, CAT bond *i* = 1, 2, …, n, ${Z}_{i}={({z}_{i1},\dots , {z}_{ip})}{\prime}$ denotes a vector of explanatory variables, namely, four macroeconomic variables, with the coefficient vector ${\theta}_{i}={({\theta}_{1},\dots , {\theta}_{p})}{\prime}$, and ${\mu}_{i}$ indicates the residual. The residual represents the pure volatility of the CAT bond price after removing macroeconomic influences. If the residual is large, it indicates that the price of the CAT bond is higher than what would be expected under macroeconomic conditions, meaning the market holds a very positive outlook for the CAT bond.

We then apply the residuals from Eq. (4) to define hard, neutral, and soft market phases based on the pooled residual distribution of all CAT and pandemic bond observations in the study sample. Soft market phases are described as the highest quintile of the residual distribution, i.e., CAT bonds trade at a premium (Hagendorff et al. 2013). Hard market phases take place in the lowest quintile of the residual distribution, i.e., CAT bonds trade at a discount. Accordingly, the neutral market is characterized by the second, third, and fourth quintiles of the residual distribution.

## Results

### Overview of CAT and pandemic bonds

#### The CAT bonds

**Fig. 1:**
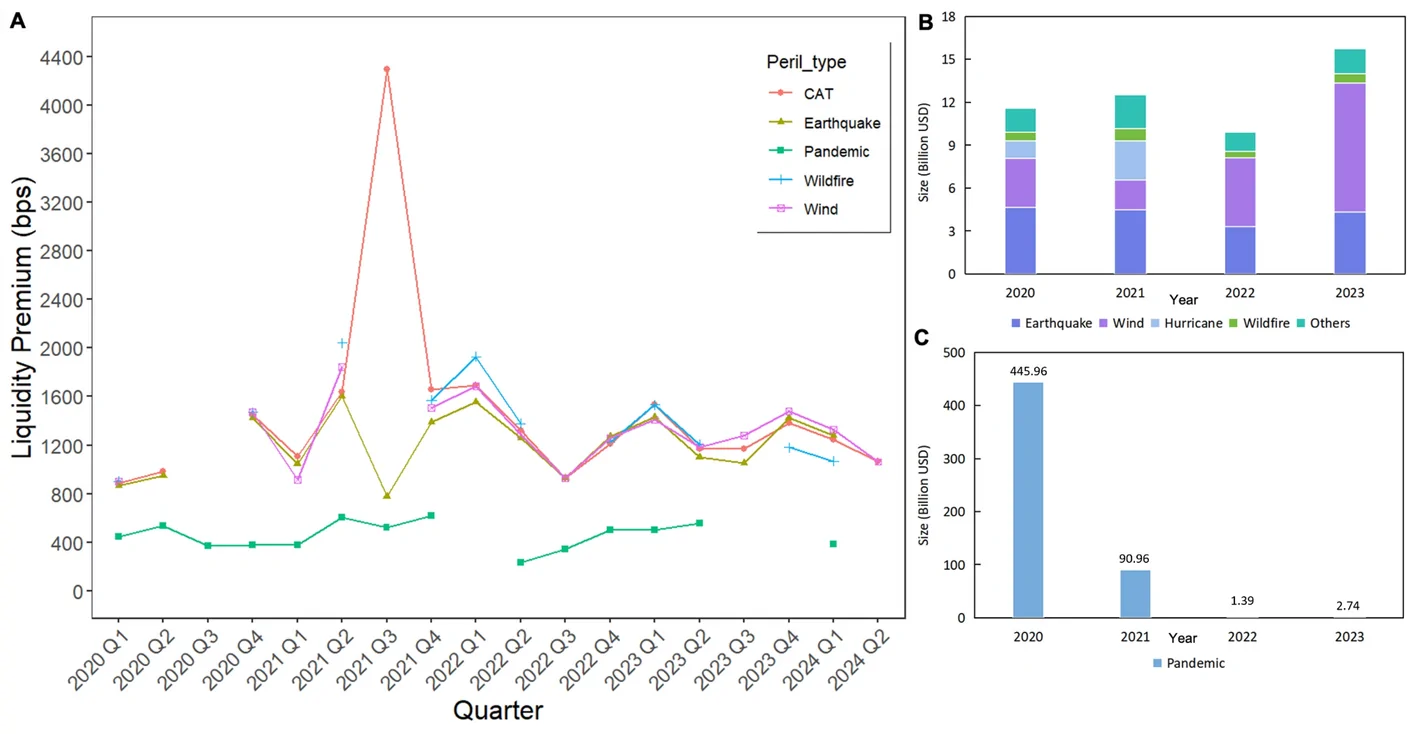
The amount and liquidity premia of CAT bonds including earthquake, wind, hurricane, wildfire, and pandemic bonds. The quarterly average liquidity premia of natural CAT bonds, earthquake bonds, pandemic bonds, wildfire bonds, and wind bonds (**A**). The yearly bond size of natural CAT bonds (**B**) and pandemic bonds (**C**) from 2020 to 2023

Natural disasters continue to impose severe human and economic burdens. In 2023, they caused a record 86,473 deaths and USD 202.7 billion in direct economic losses, exceeding the 2003–2022 annual average of USD 196 billion (Table S3). To mitigate such risks, CAT bonds and pandemic bonds have emerged as critical financial instruments, with their combined market size reaching $460 billion in 2020, accounting for 0.36% of the global bond market (Fig. 1).

Within the CAT bond sector, natural disaster-linked instruments have seen gradual growth, totaling USD 16.26 billion globally in 2023 (0.016% of the global bond market). This expansion varies by disaster type: wind-related CAT bonds dominated the market, with issuance reaching $9 billion in 2023, followed by earthquake bonds (peaking at USD 4.64 billion in 2020) and hurricane bonds (reaching USD 2.7 billion in 2021). In contrast, wildfire bonds remain a smaller segment, with a maximum size of USD 890 million in 2021, reflecting their nascent stage.

CAT bonds typically command high risk premia, with liquidity premia peaking in the first and fourth quarters, coinciding with periods of heightened disaster risk (e.g., hurricane seasons or wildfire-prone months). Natural disaster-linked CAT bonds have a higher average premium (1,364 bps) than pandemic bonds (452 bps). Among natural disaster bonds, wildfire bonds have the highest average premia (1,536 bps), followed by wind (1,382 bps), hurricane (1,373 bps), and earthquake (1,338 bps) bonds (Fig. S3).

The growing frequency and severity of wildfires, driven by climate change, have intensified financial pressures on insurers. Reinsurance capacity shortages have led to rising costs, with some regions (e.g., California’s Wildfire Fund) facing a three-year reinsurance drought (Li and Su 2024). This gap spurred innovation: 2020 marked the first issuance of California wildfire CAT bonds, initiating the market’s gradual expansion. Proceeds from these bonds are designated for practical wildfire mitigation activities, including forest management (such as removing dead trees), the development of community fire protection infrastructure, and postdisaster recovery efforts. This allocation ensures that financial instruments are directly linked to on-the-ground risk reduction.

#### Pandemic bonds

The pandemic bonds underwent striking fluctuations. With COVID-19 raging across the world in 2020, pandemic bond issuance reached USD 445.96 billion, far exceeding other types of CAT bonds. Issuance declined to USD 90.96 billion in 2021 and USD 1.39 billion in 2022 before increasing to USD 2.74 billion in 2023 and USD 6.73 billion in 2024.

Between 2020 and 2022, China was the largest issuer of pandemic bonds globally. Nonetheless, no pandemic bonds were issued in China in 2023. The total issuance of pandemic bonds amounts to USD 547.79 billion (Fig. S4), with China leading in the number of bonds issued (510) and the amount (USD 202.15 billion). SNAT follows with 47 bonds issued, totaling USD 160.59 billion. Other notable contributors include Poland, Italy, Spain, etc. Although corporations issue far more pandemic bonds than governments (619), the total size of government issuance (305 billion) is much higher than that of corporations (243 billion). Regarding bond ratings, 48% of the bonds are rated, receiving “AA” or “AAA” ratings. This suggests a high credit quality for pandemic bonds in China, which is crucial for investor confidence. The pandemic bonds in China are mainly concentrated in the more developed eastern regions. Jiangsu Province leads with the highest number of pandemic bonds issued (24), followed closely by Hubei (23). The most prevalent type of pandemic bond is private placement bonds (32.7%), while local government bonds and corporate bonds (2.4%) represent the smallest part of the pandemic bond market in China.

### The risk factors for CAT bonds

We first apply linear premium regression to examine the relationship between macroeconomic and bond-specific variables and liquidity premia. The results show that different peril locations and types exhibit substantial variation (Table 2). Macroeconomic determinants, namely, the S&P 500, Corp. Spread and term structure, are not always significant with liquidity premia. Bond-specific determinants are, by and large, more influential, especially the coupon rate and EL. Additionally, R^2^ is higher for natural CAT bonds in the premium regression. Hurricane bonds have the highest R^2^, at 0.90, followed by wildfire bonds with 0.82. Pandemic bonds have the lowest R^2^, at 0.19. In the ordinary least squares (OLS) regression for pandemic bonds, only the coupon rate and issuance market have statistically significant positive coefficients.

**Table 2:** Results of the premia regression for different kinds of CAT bonds

Variables	Hurricane	Wind	Earthquake	Wildfire	Pandemic	CAT
Constant	0.97	19.11	24.02·	17.57	-4.16	33.14***
Volume	− 2.19·	− 1.43*	− 1.24	− 1.13	0.05	− 1.31**
Maturity	2.03**	0.012	− 0.12	− 0.38	− 0.02	0.62*
Coupon	0.34	0.27**	0.38***	0.44**	1.22***	0.20**
Rating		1.94·	1.77	1.56·	0.13	− 0.31
EL	0.54	0.46*	0.51*	0.54**		0.78***
Asia/Australia	15.80***	6.53***	3.74*	41.67***		7.72***
North America	20.08***	9.39***	4.72**	− 0.96		8.69***
Europe	19.09***	11.57***	7.98***	2.38		11.08***
Other areas	26.20***	28.50***	2.86			12.10***
Hurricane						− 1.47
Wind						− 0.29
Earthquake						− 2.51***
Wildfire						2.25*
Other peril						− 0.28
S&P 500	0.003	− 0.00001	− 0.0001	0.002	− 0.0008	− 0.002*
Corp. spread	− 4.22	− 1.10	− 4.96·	1.55	1.63	− 6.25*
Term structure	0.12	− 0.045	− 0.35	0.37	1.23	− 0.33
Type					− 1.36	
Issue market					2.59**	
Obs	49	228	189	68	290	360
R^2^	0.90	0.47	0.36	0.82	0.19	0.52

(1) CAT bonds indicate natural CAT bonds. (2) Some CAT bonds cover multiple peril types. (3) All hurricane bonds are classified as ‘No rating’. (4) ·p< 0.1, * p<0.05, ** p<0.01, *** p<0.001

There are 51 CAT bonds in hard markets, 49 CAT bonds in soft markets, and another 260 in neutral markets. The CAT bond liquidity premium is higher at 1,400 bps in hard markets, followed by neutral (1,311 bps) and soft (1,278 bps) markets. Given the limited sample size, we employ the BMA to measure the determinants of CAT bonds. Overall, Bayesian regression demonstrates higher goodness-of-fit than OLS. We present the marginal posterior probabilities for all optional regressors and compare our empirical results with findings from prior studies (Table 3). There are eight risk factors with extremely high marginal posterior probabilities, mainly including different risk locations, coupon rates, ELs, earthquakes, and wildfires among risk types. We further analyze the risk factors associated with different stages of the macroeconomic cycle. European risk exposure exhibits the highest posterior inclusion support across all market phases (Table 4), indicating its consistent relevance to CAT-bond premia across market conditions. In the soft-market phase, the model with the highest posterior probability includes more credit-rating categories than the selected models for the other phases, suggesting that rating differences may help explain variation in liquidity premia within this specification (Table 5). EL is generally considered an important risk factor, and our results show that its relevance is greater in the neutral market, followed by the hard market. Higher EL is associated with higher liquidity premia in both phases, indicating greater liquidity risk.

**Table 3:** Marginal posterior probabilities of individual regressors for CAT bonds

No	Variable	PIP (%)	Effect	Prior studies
1	Asia/Australia	100	6.22	*GW, GHW*
1	North America	100	6.04	*LWT*
1	Europe	100	6.17	*GW, GHW, LWT*
4	Other area	99.99	7.04	*GW, LWT*
4	Coupon	99.99	0.28	*EUS, GHW*
6	EL	99.98	0.69	*GW, GHW*
7	Earthquake	99.60	− 2.75	*HC*
8	Wildfire	99.05	3.30	*JBW*
9	Wind	56.78	− 0.98	*GHW*
10	Volume	39.70	− 0.38	*GHW*
11	Rating 3	22.71	− 1.12	*GG, GW, GHW*
12	S&P 500	20.40	− 0.0003	*WG*
13	Corp	20.38	− 0.70	*GG, HH*
14	Maturity	17.76	0.08	*Ed, GW, KK*
15	Term	11.19	0.03	*HH, Sc*
16	Rating 2	8.11	0.13	*GG, GW*
17	Rating 4	7.72	0.23	*GG, GW, GHW*
18	Other peril type	7.03	− 0.04	*GW*
19	Hurricane	6.16	0.02	*GW, GHW*
20	Rating 5	5.69	− 0.04	*GG, GW, GHW*
21	Rating 1	5.57	− 0.09	*GG, GW*

The table also displays which variables have been found to be statistically significant in previous studies. The studies include Edwards (1986, Ed), Eom, Uno and Subrahmanyam (1998, EUS), Kamin and von Kleist (1999, KK), Weigel and Gemmill (2006, WG), Lei, Wang and Tzeng (2008, LWT), Härdle and Cabrera (2010, HC), Gürtler et al. (2016, GHW), Schwert (2017, Sc), Götze and Gürtler (2020, GG), Herrmann and Hibbeln (2021, HH), Gürtler and Witowski (2024, GW), and Jeon, Barrage and Walsh (2025, JBW). Please note that some of the variables used in prior studies are not directly equivalent to the ones used in this study and are obtained from different data sources

**Table 4:** Marginal posterior probabilities (%) of individual regressors for CAT bonds in different macroeconomic phases

Variable	Hard		Neutral		Soft	
	Effect	Prob	Effect	Prob	Effect	Prob
Volume	− 2.20	82.68	− 0.58	51.04	− 0.11	19.73
Maturity	− 0.11	20.81	0.07	15.57	− 0.04	18.16
Coupon	0.08	36.51	0.30	99.98	0.05	30.29
Rating 1			0.003	5.90	− 0.68	22.82
Rating 2	0.99	30.46	0.06	6.71	− 25.19	99.97
Rating 3			− 1.19	23.27	− 25.64	99.97
Rating 4			2.57	34.19	− 23.34	99.97
Rating 5			− 0.03	6.70	− 26.37	97.21
EL	0.50	51.06	0.46	93.36	1.17	26.67
Hurricane	0.13	19.06	0.48	20.82	− 0.31	18.58
Wind	0.19	19.46	− 0.13	12.87	0.03	20.25
Earthquake	− 0.19	20.66	− 1.59	80.63	0.15	26.90
Wildfire	0.40	22.81	0.85	35.44	0.45	18.84
Other peril type	0.19	19.64	1.75	71.76	− 0.08	18.61
Asia/Australia	− 1.18	33.79	6.29	99.95	− 0.04	31.81
North America	0.44	25.43	7.57	99.99	0.50	59.98
Europe	46.88	100.00	6.32	99.99	− 1.80	100.00
S&P 500	− 0.0002	23.95	− 0.007	9.46	0.04	0.33
Corp. spread	− 1.91	30.77	− 0.39	13.97	0.98	0.23
Term	0.21	21.44	0.004	6.74	0.23	0.26

**Table 5:** Panel regression estimates of the best model for CAT bonds with the highest posterior probabilities

Variable	CAT	Soft	Neutral	Hard
Intercept			6.65***	6.85
Maturity	1.37***		− 0.06	0.98
Rating 1		− 4.95		
Rating 2		8.62		
Rating 3		7.81		
Rating 4		− 1.45		
Rating 5	− 14.11**		− 3.48	
Hurricane	− 0.79			
Wind	− 1.37·		− 1.69	
Earthquake	− 2.58***			
Other peril type	2.43**		3.97***	
Asia/Australia	6.47***		4.96***	
North America	10.56***	12.93***	7.52***	4.64
Europe	0.41***	15.56***	6.62***	
R^2^	0.83	0.79	0.86	0.74
Prob	11.00	1.69	4.63	1.65

^***^, **, * and ^.^ indicate significance at the 0.1%, 1%, 5% and 10% levels

### Risk factors for pandemic bonds

#### Pandemic bonds in different macroeconomic stages

There are 101 pandemic bonds in the hard market, 54 in the soft market, and 135 in the neutral market. Similarly, the pandemic bond liquidity premium is higher at 517 bps in hard markets, followed by neutral (451 bps) and soft (425 bps) markets. Coupon rates have relatively high posterior inclusion probabilities across all market phases (Table 6). Support for issuance in nondomestic markets varies across phases, with greater inclusion probabilities in soft and neutral markets than in hard markets. In the soft-market subsample, several predictors receive comparatively high posterior inclusion support. The S&P 500 index has the highest marginal a posteriori probability (83.69%) in explaining liquidity premia, followed by issuance in nondomestic markets, coupon rate, Rating 3, and maturity. According to the best Bayesian regression in Table S4, the goodness of fit is higher in the soft market. In the selected soft-market model, issuance in nondomestic markets is associated with a 166-bps higher estimated premium than domestic issuance. A one-year increase in maturity corresponds to a 5-bps higher estimated premium, conditional on the other variables in the model.

**Table 6:** Marginal posterior probabilities (%) of individual regressors for pandemic bonds in different macroeconomic phases

Variable	Pandemic		Soft		Neutral		Hard	
	Effect	Prob	Effect	Prob	Effect	Prob	Effect	Prob
Coupon	1.25	99.99	0.25	71.79	1.65	99.99	1.10	97.86
Issue market	2.53	94.07	0.61	73.46	4.41	97.35	0.03	9.67
Type	− 0.32	22.01	− 0.17	34.37	− 0.76	31.08	− 0.29	16.47
Rating 3	− 0.34	18.81	− 1.00	67.76	− 0.54	21.22	− 0.11	10.63
Term	0.15	13.74	0.19	30.55	0.75	37.91	− 0.03	9.48
Maturity	− 0.002	7.54	0.03	61.33	− 0.002	9.15	− 0.002	10.14
Volume	− 0.01	7.43	− 0.009	26.53	− 0.23	31.59	0.04	11.84
Rating 6	0.04	6.59	0.19	35.13	0.06	8.82	0.03	9.20
Rating 2	− 0.09	6.46			− 0.01	7.58	− 0.17	10.20
Rating 4	− 0.02	6.30	− 0.15	32.84	− 0.009	8.51	− 0.21	12.99
Rating 5	− 0.03	6.04	− 0.24	35.45	0.03	8.79	− 0.04	9.46
Corp. spread	0.04	6.02	1.36	37.83	0.08	9.60	0.16	11.01
S&P 500	− 0.005	6.00	0.00002	83.69	0.005	9.50	− 0.003	11.76

Effect denotes the model− averaged coefficient; Prob denotes the posterior inclusion probability (%)

#### The influence of ESG score on pandemic bonds

In this study, Bloomberg ESG scores are available for 95 pandemic bonds. Their average premium is 439 bps, slightly lower than that of bonds without ESG scores. Given the limited ESG-covered sample, we use bootstrap resampling to assess uncertainty in the estimated relationships between bond characteristics and premia. Table 7 reports the results based on 1,000 bootstrap resamples. Based on the 95% CI calculated using the BCA (bias-corrected and accelerated) bootstrap method, variables are considered statistically significant if their confidence intervals do not contain 0 (*p* < 0.05). A one-percentage-point increase in the coupon rate corresponds to a 0.90-percentage-point higher estimated premium (95% CI [0.20, 2.79]). A one-year increase in maturity is associated with a 26 bps higher premium (95% CI [0.4, 135]). The estimated coefficient for the ESG score is positive but statistically insignificant. This suggests that the liquidity premia of pandemic bonds within this subsample do not clearly reflect aggregate ESG performance. One possible explanation for this is the limited variation in ESG scores, given that most issuers are clustered around medium levels. Additionally, the relatively small sample size and generic nature of composite ESG indicators may constrain their explanatory power in the context of pandemic-specific financial instruments.

**Table 7:** The comparison of OLS and Bootstrap for pandemic bonds with ESG

Variable	OLS estimate	OLS Sd	Bootstrap estimate	Bootstrap Sd error	Bootstrap bias	Lower 95% CI	Upper 95% CI	Significant
Volume	0.35	0.48	0.31	0.47	− 0.04	− 0.19	1.96	No
Maturity	0.19	0.12	0.26	0.28	0.07	0.004	1.35	Yes
Coupon	0.90	0.25	0.90	0.57	0.002	0.20	2.79	Yes
Type	0.72	1.38	0.69	0.99	− 0.04	− 0.43	4.58	No
Rating	0.21	0.27	0.18	0.21	− 0.03	− 0.59	0.44	No
Issue market	1.24	1.30	1.21	0.92	− 0.03	− 1.42	2.53	No
S&P 500	− 0.004	0.004	− 0.003	0.004	0.0003	− 0.02	0.001	No
Term	3.25	2.64	3.03	2.76	− 0.22	− 0.05	14.23	No
Corp. spread	− 1.47	4.28	− 1.27	2.64	0.21	− 10.36	1.84	No
ESG Score	0.003	0.04	0.003	0.03	− 0.0003	− 0.04	0.07	No

#### Health score-augmented BMA results

To further incorporate pandemic-specific health-system characteristics into the empirical analysis, we introduce the health-specific index into the full pandemic bond sample within the BMA framework. We also compare models including ESG scores alone and jointly with health-score categories in the same 95-bond ESG-covered subsample. Pandemic bonds with lower health scores exhibit the highest average premia (555 bps), while average premia are lower for medium-score bonds (405 bps) and lowest for high-score bonds (382 bps). This pattern indicates a clear gradient across health score groups. The distribution of issuers also varies across health score groups. Low-score bonds are predominantly corporate issuances, and government issuers account for a much larger share of high-score bonds (Fig. S5).

Health-score categories have negative model-averaged coefficients in both specifications that include them, although posterior inclusion support differs across samples (Table 8). In the full sample, the high- and medium-health-score categories have coefficients of − 0.02 and − 0.008 and inclusion probabilities of 6.67% and 5.63%, respectively. In the ESG-covered subsample with both ESG and health score variables, the corresponding coefficients are − 0.15 and − 0.40, with inclusion probabilities of 45.19% and 49.74%, respectively. All comparisons use the low-health-score category as the reference. This pattern is consistent with the lower risk compensation required by investors for bonds linked to issuers with stronger disclosed health-related capacity.

**Table 8:** Marginal posterior probabilities (%) of individual regressors for pandemic bonds with ESG score and health-specific index

Variable	Model with health score		Model with ESG score		Model with ESG and health scores	
	Effect	Prob	Effect	Prob	Effect	Prob
Coupon	0.89	99.99	0.13	53.13	0.12	53.65
Issue market	0.08	53.28	0.35	48.10	0.34	48.62
Type	− 0.05	8.21	− 0.18	45.19	− 0.15	45.17
Rating 3	− 0.04	22.69	− 0.40	46.37	− 0.41	47.24
Term	0.04	7.38	− 0.23	44.74	− 0.16	45.52
Maturity	0.00007	5.82	− 0.01	44.12	− 0.01	44.89
Volume	0.02	9.70	0.10	46.74	0.10	47.32
Rating 6	0.02	17.18	− 0.25	47.07	− 0.30	48.00
Rating 2	− 0.03	5.97				
Rating 4	− 0.02	6.42	0.11	44.55	0.05	45.53
Rating 5	0.10	11.35	− 0.56	51.77	− 0.59	52.67
Corp. spread	0.01	6.40	1.45	55.21	1.37	54.72
S&P 500	− 0.003	7.43	0.01	47.90	0.002	48.25
ESG score			− 0.001	42.78	− 0.002	44.76
High health score	− 0.02	6.67			− 0.15	45.19
Medium health score	− 0.008	5.63			− 0.40	49.74

All models include the baseline bond-specific and macroeconomic predictors. The first model adds health score categories to the full sample of 290 pandemic bonds. The second includes ESG scores, and the third jointly includes ESG scores and health-score categories, both using the same 95-bond ESG-covered subsample. The low-health-score category is the reference group. Rating 2 is absent from both 95-bond models because none of these bonds belongs to that category. Blank cells indicate that the variable is not included or the category is absent from the sample

The ESG score also has a negative coefficient in both models that include it. In the ESG-only specification, its coefficient is approximately − 0.001, with a posterior inclusion probability of 42.78%. When jointly included with health-score categories, the coefficient is − 0.002, and the inclusion probability is 44.76%. Within the joint specification, the inclusion probabilities of the high- and medium-health-score categories are slightly higher than that of ESG and broadly comparable to those of several conventional bond characteristics, including maturity, term structure, and issue volume.

Including the health-specific index does not change the fact that coupons remain among the predictors with the highest posterior inclusion probabilities across the specifications. Overall, these results suggest that issuers’ disclosed health-related capacity, as captured by the health-specific index, may be associated with pandemic bond pricing. However, given the limited ESG-covered subsample and the early stage of the pandemic bond market, the estimated effects of the health index should be considered indicative rather than definitive.

### Robustness check

#### Results of the SHAP

We further employ IML to identify the macroeconomic and bond-specific factors of CAT bond liquidity premia. First, six ML techniques are applied to premia regression. The full sample is divided into a training set (60%) and a held-out test set (40%) for model evaluation. The evaluation metrics encompass R^2^, mean squared error (MSE), mean absolute error (MAE), and root mean square error (RMSE), where R^2^ is the primary metric. Among the test data of six models, XGBoost (extreme gradient boosting) achieved the highest R^2^ of 0.65, explaining 65% of the variance in bond premia, while MLP (multilayer perceptron) had the lowest R^2^ at 0.34, indicating poor data fit. For MSE, DT (decision tree) performed worst with an MSE of 185.26, whereas GBRT (gradient boosting regression tree) and XGBoost showed superior accuracy with lower MSE values (19.58 and 19.76, respectively). The RMSE followed a similar pattern: DT had the highest RMSE (13.61), while XGBoost and GBRT had lower values (4.44 and 4.43). MAE further validated XGBoost's advantage, with the model demonstrating its ability to predict average error with greater accuracy. DT had the highest MAE (12.44), and XGBoost had the lowest (2.85). Overall, XGBoost achieved the strongest predictive performance within our empirical setting, making it the preferred model for predicting bond premia (Fig. S6).

**Fig. 2:**
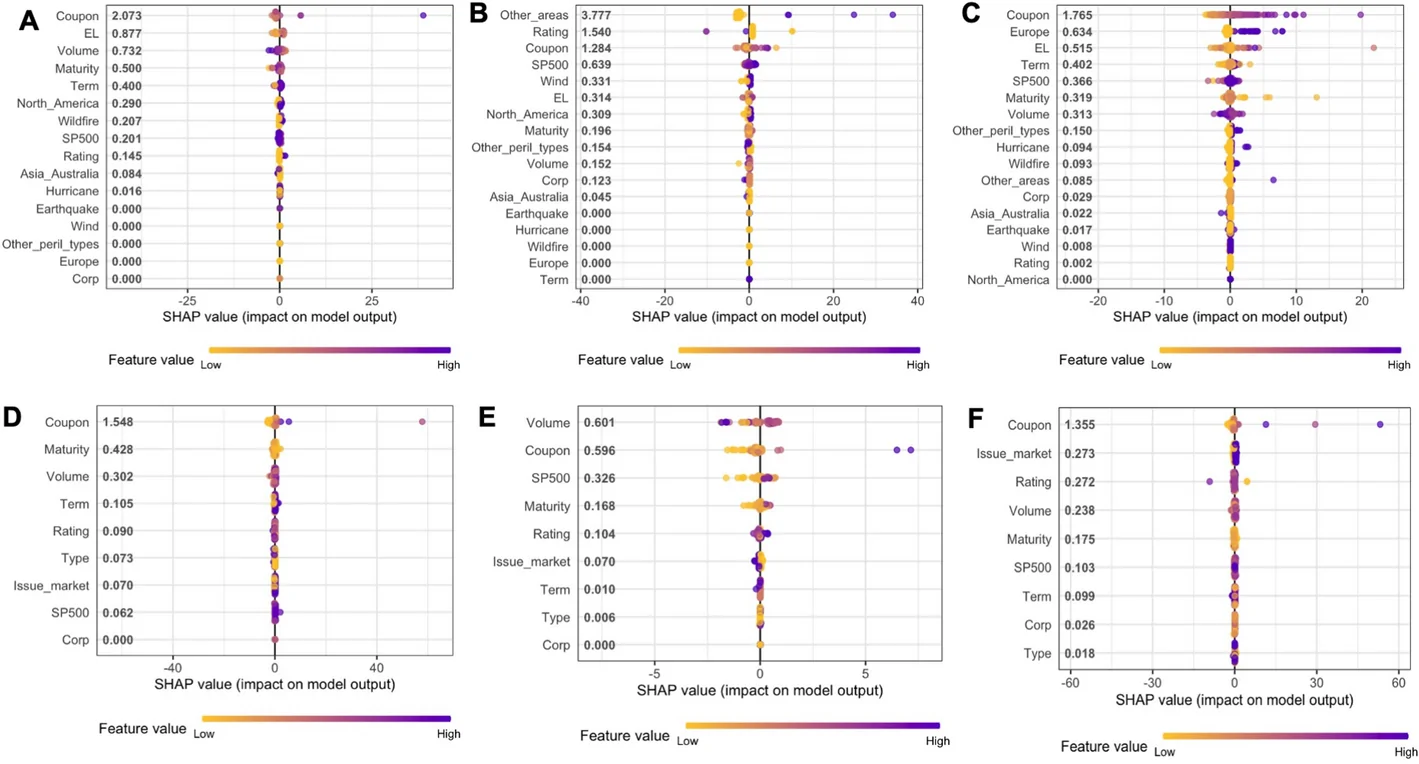
SHAP summary plots of liquidity premia for CAT bonds in different market phases. The hard market (**A**), soft market (**B**), and neutral market (**C**) for natural CAT bonds, and the hard market (**D**), soft market (**E**), and neutral market (**F**) for pandemic bonds

The SHAP (SHapley Additive exPlanations) method is employed to visualize and interpret the contribution of each liquidity risk factor to model predictions, providing a transparent understanding of variable importance (Yao et al. 2024). The SHAP, coupled with XGBoost, is further used to compare differences in the main determinants of CAT bond premia across different markets. Figure 2 shows that the drivers of the liquidity premium in a neutral market are essentially unchanged when there is no defined market cycle. Some variation is observed in hard markets, with European markets and Corp. spreads making smaller contributions to the model’s predictions of liquidity premia and bond-related factors accounting for a larger contribution. In soft markets, however, the drivers change dramatically. This indicates that bonds covering a broader range of risk-exposed regions tend to have lower market liquidity and higher bond premia. At higher overall bond market premia, bond credit ratings become more prominent in the model, suggesting greater market attention to default risk. The overall SHAP values of risk factors for CAT bonds further validate the BMA conclusions.

Pandemic bonds are more susceptible to different market phases, notably in the soft market. Issue volume is the most influential factor, followed by coupon rate, S&P 500, maturity, rating, issue market, term structure, and type. These results highlight the importance of issue volume in assessing pandemic bond liquidity risk during soft market periods. For the hard market, the main determinants are, in order, coupon rate, maturity, volume, term structure, rating, type, issue market, S&P 500, and Corp. spread. In a neutral market, the coupon rate is also the primary driver. The overall SHAP values of risk factors for pandemic bonds further validate the BMA's findings.

#### Results of the synthetic data for pandemic bonds

To validate the robustness of the conclusions drawn from the data in Table 6, we used synthetic data to demonstrate the marginal posterior probabilities and effect sizes of pandemic bonds across different macroeconomic phases. This method is used to test model stability during simulated data generation. All synthetic data were benchmarked against the distribution characteristics of the original bond data to avoid introducing fictitious information detached from real-world operations. An additional 2% noise was applied to numerical variables. First, the core variables demonstrate consistently high explanatory power across both tables. Table 6 reports very high posterior probabilities for the coupon rate (e.g., 99.99% in neutral phases and 97.86% in hard phases), and Table 9 also shows high probabilities for the coupon rate (100.00% in neutral phases, 99.95% in hard phases, and 64.87% in soft phases). These results further support the robust role of the coupon rate in explaining pandemic bond dynamics, with aligned effect directions. Second, variables such as the issue market and maturity exhibit coherent patterns: the issue market reaches a high probability of 99.85% in the neutral market phase, which is consistent with the high probability of 97.35% reported in Table 6 for the same phase. Maturity receives relatively high posterior inclusion support in both the original and synthetic soft-market samples, at 61.33% and 92.71%, respectively. Overall, the synthetic-data results preserve the positive coupon association across market phases and the strong inclusion support for the issuance market in the neutral phase.

**Table 9:** Marginal posterior probabilities (%) for pandemic bonds in different macroeconomic phases with synthetic data

Variable	Hard		Neutral		Soft	
	Effect	Prob	Effect	Prob	Effect	Prob
Volume	0.27	53.06	− 0.19	55.59	− 0.001	17.43
Maturity	− 0.008	2.33	− 0.003	24.88	− 0.04	92.71
Coupon	1.20	99.95	0.93	100.00	0.16	64.87
Rating 2	− 0.83	36.81	0.007	23.69		
Rating 3	− 0.41	34.91	− 1.31	70.58	− 1.65	97.28
Rating 4	− 0.49	39.06	0.02	24.10	− 0.09	23.76
Rating 5	− 0.08	28.53	− 0.08	25.64	− 0.56	62.12
Rating 6	− 0.09	28.64	0.18	28.23	0.03	17.67
Type	− 0.72	50.24	− 0.38	40.49	− 0.24	40.65
Issue market	0.05	30.45	3.00	99.85	0.41	51.68
S&P 500	− 0.0004	32.63	0.0003	29.38	0.004	99.98
Corp. spread	0.08	30.74	0.38	29.22	1.13	34.75
Term	− 0.02	29.39	0.64	62.67	− 0.09	18.15

#### Liquidity premia across macroeconomic phases for pandemic bonds

The initial COVID-19 outbreak may have coincided with unusual issuance and market conditions in early 2020. As a robustness check, we re-estimate the BMA models after excluding pandemic bonds issued in the first quarter of 2020 (Table 10). The first two specifications use the same restricted sample, with and without market-phase indicators. The remaining specifications report phase-specific results. In the specification with market-phase dummies, neutral market conditions serve as the reference category, with separate dummy variables for hard and soft markets.

**Table 10:** Marginal posterior probabilities (%) of individual regressors for pandemic bonds across macroeconomic phases, excluding bonds issued in the first quarter of 2020

Variable	With market-phase indicators		Without market-phase indicators		Soft		Neutral		Hard	
	Effect	Prob	Effect	Prob	Effect	Prob	Effect	Prob	Effect	Prob
Coupon	1.28	99.99	1.29	99.98	0.37	84.00	1.44	99.98	0.60	88.61
Issue market	2.82	99.09	2.72	96.37	0.73	62.53	4.48	95.81	0.004	39.48
Type	− 0.68	53.38	− 0.83	56.92	− 0.12	31.23	− 0.95	48.17	− 0.37	44.71
Rating 3	− 1.19	61.11	− 0.92	50.21	− 0.98	63.87	− 1.04	41.92	− 0.23	39.99
Term	0.60	50.99	1.03	61.11	− 0.67	34.81	1.43	61.18	0.10	39.63
Maturity	− 0.006	32.03	− 0.006	32.11	0.02	49.04	− 0.007	30.22	− 0.003	39.21
Volume	− 0.05	31.32	− 0.07	33.41	0.01	29.17	− 0.43	58.33	0.09	43.19
Rating 6	0.09	28.51	0.10	28.02	0.31	42.91	0.06	30.68	− 0.05	39.46
Rating 2	− 0.57	32.73	− 0.59	32.06			− 0.008	28.52	− 0.36	40.84
Rating 4	− 0.11	29.62	− 0.22	32.65	− 0.27	41.38	− 0.37	33.64	− 0.39	43.42
Rating 5	− 0.21	31.42	− 0.18	30.41	− 0.08	28.99	0.29	31.58	− 0.12	39.39
Corp. spread	0.32	31.23	1.98	51.73	0.18	31.30	4.93	46.03	0.19	39.75
S&P 500	0.00002	28.71	0.0001	34.37	0.002	67.50	0.002	41.94	− 0.0003	40.68
Hard	0.45	45.28								
Soft	− 0.03	28.10								

All specifications exclude pandemic bonds issued in the first quarter of 2020. The first two specifications use the same restricted sample, with and without market-phase indicators

Across all specifications, coupon rates remain the most robust determinant of liquidity premia, retaining high posterior inclusion probabilities in the pooled and phase-specific specifications. The issuance market and Rating 3 also receive appreciable posterior inclusion support in the pooled specifications and the soft-market subsample, while support for the issuance market remains particularly high in the neutral-market subsample. Bond maturity and term structure variables display relatively lower inclusion probabilities; however, their signs are largely consistent with the baseline estimates in Table 6.

In the restricted sample, selected macroeconomic variables receive greater posterior inclusion support than in the baseline analysis. When market-phase dummies are included in the pooled regression, the hard-market coefficient is positive, with a posterior inclusion probability of 45.28%, suggesting a higher estimated liquidity premium relative to neutral market conditions. In contrast, the soft market dummy has a small negative coefficient and a lower inclusion probability. Comparing Tables 6 and 10, the positive coefficients for the coupon and issuance markets are preserved across market phases. Overall, the consistency of the main coefficient signs and predictor rankings after excluding early-2020 observations supports the robustness of the principal findings to this sample restriction.

#### Maturity and SOFR for pandemic bonds

Replacing synthetic LIBOR with SOFR-based rates preserves the positive model-averaged coefficients for coupon and issuance in nondomestic markets, consistent with the direction of the baseline associations (Table 11, “SOFR” columns). Credit-rating variables and term structure receive greater posterior inclusion support under the SOFR-based specification, while support for the coupon and issuance market is lower. Thus, the alternative benchmark retains the direction of these two baseline associations, although the relative importance of individual predictors varies with the benchmark used.

**Table 11:** Marginal posterior probabilities (%) of individual regressors for pandemic bonds under maturity restrictions and an alternative benchmark rate

Variable	Baseline		Short maturity		SOFR	
	Effect	Prob	Effect	Prob	Effect	Prob
Coupon	1.25	99.99	1.32	99.99	0.02	27.36
Issue market	2.53	94.07	2.89	98.75	0.34	30.66
Type	− 0.32	22.01	− 0.64	50.53	− 2.20	58.66
Rating 3	− 0.34	18.81	− 1.27	59.55	− 3.65	62.56
Term	0.15	13.74	0.77	56.60	− 2.63	66.50
Maturity	− 0.002	7.54	− 0.008	29.00	− 0.09	55.07
Volume	− 0.01	7.43	− 0.04	30.25	− 0.04	28.09
Rating 6	0.04	6.59	0.09	28.02	− 4.23	65.78
Rating 2	− 0.09	6.46	− 0.56	32.32	− 1.15	33.45
Rating 4	− 0.02	6.30	− 0.14	30.55	− 5.41	80.30
Rating 5	− 0.03	6.04	− 0.24	32.33	− 7.48	65.78
Corp. spread	0.04	6.02	0.30	31.28	2.67	44.68
S&P 500	− 0.005	6.00	− 0.005	28.75	0.0003	32.94

Baseline refers to the full-sample pandemic bond specification reported in the main analysis. Short maturity excludes bonds with maturities exceeding 30 years. SOFR uses SOFR-adjusted benchmark rates in place of the original LIBOR-based benchmark

In addition, we perform a robustness check by excluding ultralong-maturity pandemic bonds with maturities above 30 years (Table 11, “Short maturity” columns). The BMA results show that core pricing variables exhibit consistent coefficient signs, magnitudes, and posterior inclusion probabilities across the full and restricted samples. The coupon rate and issuance market retain positive coefficients and high posterior inclusion probabilities after excluding ultralong-maturity bonds. Bond type, selected rating categories, and term structure receive greater inclusion support in the restricted sample. Overall, the main findings remain qualitatively unchanged after excluding ultralong-maturity observations.

#### Subsample analyses by issuer location and currency

The pandemic bond sample includes issuers from different institutional settings and instruments denominated in multiple currencies, while the macroeconomic benchmarks are largely US-centered. Therefore, we conduct separate BMA analyses for China, non-China, and USD-denominated instruments to assess whether the baseline findings are sensitive to issuer location and currency composition.

In the China and non-China subsamples, coupon rates remain the leading predictor, with positive coefficients of 1.58 and 0.85 and posterior inclusion probabilities of 99.98% and 99.93%, respectively (Table 12). These results are consistent with the full-sample coefficient of 1.25 and inclusion probability of 99.99%. Issuance in nondomestic markets also retains a positive coefficient in both subsamples. In the China subsample, its coefficient is 4.49, with a posterior inclusion probability of 97.37%. In the non-China subsample, the coefficient is 0.26, and the posterior inclusion probability is 18.30%, placing it second after the coupon despite the relatively low absolute inclusion probability. The corresponding baseline coefficient and inclusion probability are 2.53 and 94.07%, respectively. Overall, the positive association between coupon and premium receives consistently strong support in both subsamples, indicating that it is not confined to Chinese issuances. Support for issuance in nondomestic markets is concentrated in the China subsample.

**Table 12:** Marginal posterior probabilities (%) of individual regressors for pandemic bonds by issuer location and currency

Variable	Baseline		China		Non-China		USD	
	Effect	Prob	Effect	Prob	Effect	Prob	Effect	Prob
Coupon	1.25	99.99	1.58	99.98	0.85	99.93	0.40	70.59
Issue market	2.53	94.07	4.49	97.37	0.26	18.30	0.17	45.68
Type	− 0.32	22.01	− 1.56	61.00	− 0.08	9.85	− 0.25	45.58
Rating 3	− 0.34	18.81	− 0.98	42.10	− 0.07	8.38	− 0.15	43.85
Term	0.15	13.74	1.15	61.71	0.006	7.32	0.12	43.09
Maturity	− 0.002	7.54	− 0.01	34.58	− 0.00009	6.81	− 0.006	43.51
Volume	− 0.01	7.43	− 0.30	47.83	0.02	9.77	0.11	47.51
Rating 6	0.04	6.59	− 0.16	27.06	0.05	7.88	− 0.02	45.51
Rating 2	− 0.09	6.46	− 0.80	31.22	− 0.01	6.87	− 0.32	44.49
Rating 4	− 0.02	6.30	0.03	27.53	− 0.06	8.57	− 0.26	45.05
Rating 5	− 0.03	6.04	0.33	30.91	− 0.09	9.38	− 0.12	44.02
Corp. spread	0.04	6.02	− 0.47	33.73	0.13	9.97	0.86	46.37
S&P 500	− 0.005	6.00	0.03	32.83	− 0.004	8.62	0.01	44.00

Baseline refers to the full-sample pandemic bond specification reported in the main analysis. China and non-China refer to subsamples of bonds issued by issuers located in China and outside China, respectively. USD restricts the sample to US dollar-denominated bonds

In the USD-denominated subsample, the coupon remains the leading predictor, with a positive coefficient of 0.40 and a posterior inclusion probability of 70.59% (Table 12). Issuance in nondomestic markets also retains a positive coefficient of 0.17, with an inclusion probability of 45.68%. Thus, the positive coefficient signs of both baseline predictors and the leading position of the coupon are preserved when the sample is restricted to USD-denominated instruments, although their posterior inclusion probabilities are lower than in the full sample.

#### Evidence from green bonds

Given the small sample size of pandemic bonds with ESG scores, the generalizability of the findings is limited. We further collected 1,943 green bonds from Bloomberg to provide comparative evidence. From 2020 to 2023, green bonds in our sample have an average estimated liquidity premium of 330 bps, lower than that of pandemic bonds. The BMA results suggest that Corp. spread is the most critical risk factor, followed by ESG scores (Table 13). A one-unit increase in the ESG score is associated with a 0.4 bp increase in the risk premium of green bonds. Although small in magnitude, this positive association is consistent with prior evidence that ESG ratings often operate through an information channel rather than a pure risk-reduction mechanism (Avramov et al. 2022). Prior studies suggest that rating divergence and potential greenwashing may complicate investors’ interpretation of ESG information. In settings with high information asymmetry, opaque or inconsistent ESG signals can increase perceived uncertainty, leading investors to demand additional compensation that includes a liquidity premium component. When ESG signals are instead credible and transparent, investors are more willing to accept lower yields (Baldi and Pandimiglio 2022; Zou et al. 2023). This contrast is consistent with the view that ESG metrics play a clearer role in the more mature green bond market but offer weaker and less informative signals for pandemic bonds, where health-specific measures may capture pricing associations not fully reflected in ESG scores.

**Table 13:** Marginal posterior probabilities of individual regressors for green bonds with ESG scores

No	Variable	Effect	Prob (%)
1	Corp. spread	− 1.53	100.00
2	ESG score	0.004	99.92
3	Coupon rate	0.41	99.77
4	Maturity	0.003	99.73
5	Volume	− 0.21	99.69
6	Term	0.25	99.65
7	S&P 500	− 0.0005	99.61

### Explanation for heterogeneity

**Fig. 3:**
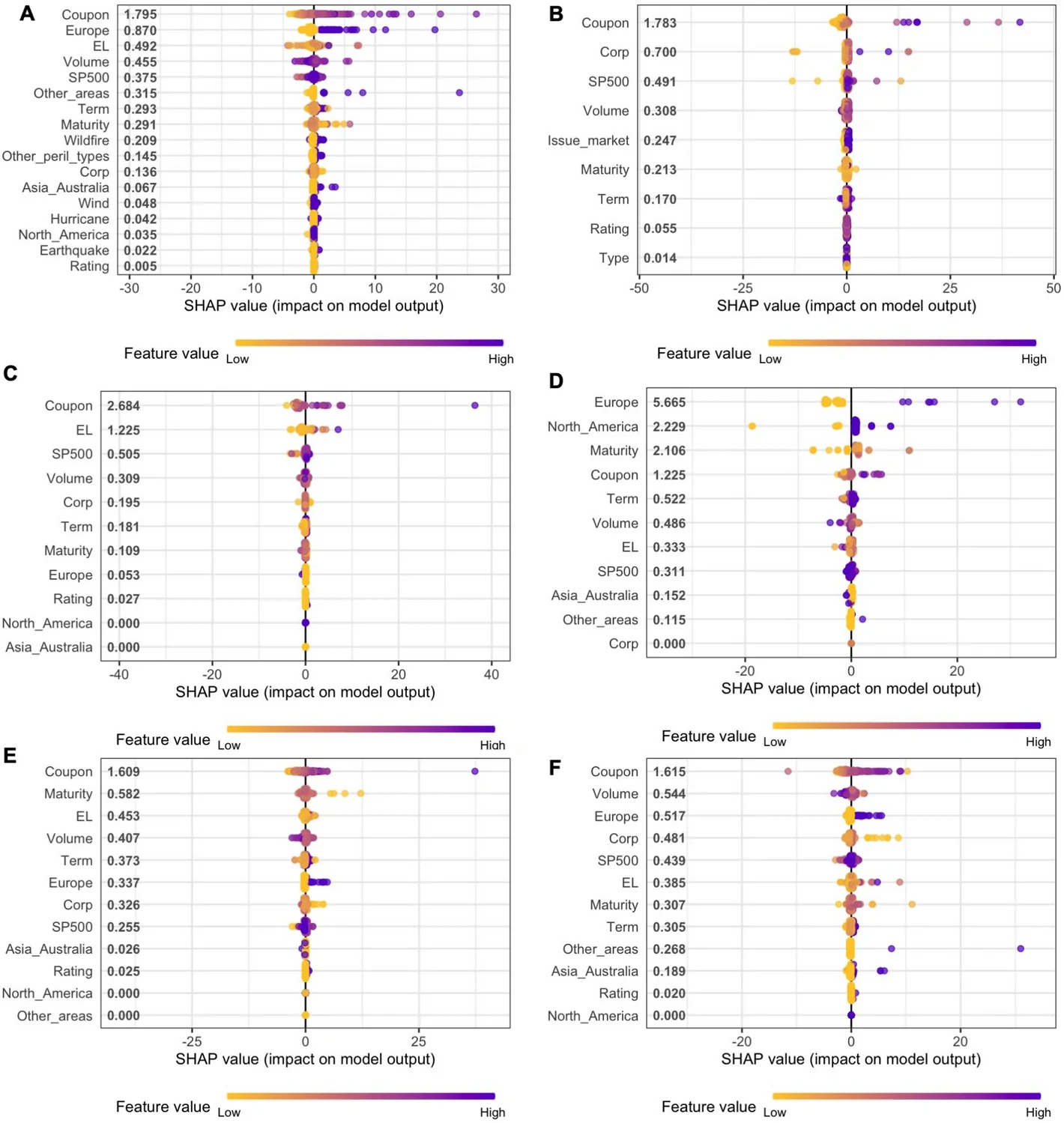
SHAP summary plots of each kind of CAT bond. Natural CAT bonds (**A**), pandemic bonds (**B**), wildfire (**C**), hurricane (**D**), earthquake (**E**), and wind (**F**). The numbers on the left in the table denote the SHAP values

We further explain the contribution of liquidity risk factors for different types of CAT bonds using the SHAP technique. The results show that for almost all types of CAT bonds, the coupon rate plays a positive and dominant role in the liquidity premium (Fig. 3). Higher coupon rates generally reflect greater compensation required by investors for bearing higher levels of risk, which is associated with higher liquidity premia. Other bond-specific drivers are, in order, EL, trading volume, maturity, and bond ratings. Higher EL is associated with higher liquidity premia, while greater bond volume tends to be linked to lower liquidity premia. Short-term bonds are generally more liquid. Bonds with high credit ratings are generally more liquid, as investors perceive them to have a lower risk of default. However, credit ratings have relatively low SHAP importance in our analysis, possibly because most bonds in the sample are unrated. Regarding hazard location and type, Europe and wildfires rank higher in SHAP importance, while North America and earthquakes rank lower. Among macroeconomic factors, the S&P 500 has the highest SHAP importance, followed by term structure and Corp. spreads. Overall, macroeconomic factors generally exhibit positive associations with liquidity premia.

Next, we further compare the liquidity risk factors across different CAT bond types. Whether hurricane bonds cover risks in Europe or North America is particularly relevant to their predicted liquidity premia, as these geographical indicators have the highest SHAP importance in the model (Fig. 3). This pattern may reflect regional differences in natural hazard characteristics and frequency. For most other natural CAT bond types, coupon rates remain the dominant determinant. Pandemic bonds are considerably different. Bonds issued outside the domestic market carry a higher premium than those issued locally. Premia for government-issued bonds is higher than that for corporate-issued bonds.

## Discussion

Pandemic bonds are exposed to liquidity-related risks that may vary across market phases. Between 2020 and 2024, their estimated liquidity premium averaged 452 bps in our sample. In the soft-market subsample, the S&P 500 index has the highest posterior inclusion probability (83.69%), followed by issuance in nondomestic markets, coupon rates, Rating 3, and maturity. These results highlight the relevance of both macroeconomic conditions and bond characteristics during soft markets. Estimates from the selected soft-market model further associate nondomestic issuance and longer maturity with higher premia. These findings suggest that issuers should closely monitor macroeconomic indicators during soft markets and consider the potential financing cost implications of nondomestic issuance and longer maturities when structuring bonds.

The market-phase differences observed in our analysis can be considered alongside the broader literature on insurance and reinsurance cycles. In the markets for insurance, reinsurance and ILS, it is well established that pricing dynamics vary systematically across market cycles, reflecting fluctuations in the supply of risk-bearing capital (Gürtler et al. 2016; Hagendorff et al. 2013). In particular, prior studies have shown that, during hard markets, underwriting characteristics and capital constraints play a dominant role. In contrast, in soft markets, abundant capital compresses spreads and reduces the sensitivity of bond pricing to detailed risk characteristics (Götze and Gürtler 2020). Our findings extend these studies by documenting phase-dependent pricing associations in the pandemic-bond market.

The variation in predictor importance across market phases highlights the need to account for model uncertainty when examining pandemic-bond liquidity premia. Macroeconomic conditions, bond characteristics, and sustainability-related information represent different dimensions potentially associated with pricing, and the evidence for their inclusion may vary across market phases. Relying on a single selected regression specification may therefore provide an incomplete account of these associations. BMA provides a coherent framework for addressing this uncertainty by averaging across alternative combinations of predictors and accounting for their posterior model probabilities (Maltritz and Molchanov 2013). For the relatively underdeveloped pandemic bond market, this approach provides exploratory evidence on which pricing associations persist across market phases and which are more dependent on market conditions.

Disclosure of ESG factors remains relatively limited among pandemic bonds. In our sample, bonds with available ESG scores exhibit slightly lower average liquidity premia than those without ESG scores. ESG scores have negative model-averaged coefficients in both BMA specifications that include them, with posterior inclusion probabilities of 42.78% and 44.76%. Compared with the stronger posterior inclusion support for ESG in our green bond analysis, these results suggest that the pricing relevance of ESG scores differs across the two bond samples. Generic ESG measures may capture only part of the information relevant to pandemic-bond pricing. To examine health-related characteristics more directly, we incorporate a disclosure-based health-specific index. Higher health-score categories have negative model-averaged coefficients relative to the low-score category in both specifications that include them. In the joint specification, these categories receive slightly greater posterior inclusion support than ESG. This pattern suggests that disclosed health-related characteristics may contain information relevant to pandemic-bond pricing, although the moderate inclusion probabilities and limited sample size warrant a tentative interpretation. These findings motivate further consideration of health-specific disclosures alongside conventional ESG measures, including health system capacity, public health surveillance and emergency preparedness, public financing commitments to health, and health-aligned use of proceeds and reporting. These dimensions can be viewed as an exploratory step toward an ESHG-type (environmental, social, health and governance) sustainability framework tailored to health-linked financial instruments.

Beyond sustainability-related information, the natural CAT-bond results highlight the relevance of peril-specific risk characteristics to pricing. These findings underscore the importance of accounting for catastrophe-specific risks in bond pricing, particularly given the role of CAT bonds in financing catastrophe-related losses despite their small share of the global bond market. Wildfire bonds provide an example of instruments designed to transfer specific catastrophe risks to capital markets, with funds allocated to forest management and disaster recovery. In our sample, they carry the highest average premium among the peril categories examined, at 1,536 bps. Expected loss and regional risk exposure are among the factors associated with their premia (Table S5).

Turning to the comparison between natural CAT and pandemic bonds, pandemic bonds have a lower average premium than natural CAT bonds in our sample (452 versus 1,364 bps). The observed premium gap coincides with differences in the characteristics of the two bond samples, including lower average coupon rates for pandemic bonds (Table 1). Coupon has a positive coefficient and a high posterior inclusion probability in both BMA models (Tables 3 and 6), and the SHAP results also identify it as an important predictor across bond types (Fig. 3). These findings suggest that differences in bond characteristics may help account for the observed premium gap, but the cross-sample comparison does not establish a causal explanation. Differences in risk assessment and contractual design may also be associated with differences in premia. The more established risk-modeling frameworks used for natural CAT bonds provide explicit measures of catastrophe exposure and expected loss. Pandemic-related instruments encompass different contractual structures. For example, the World Bank’s PEF bonds comprise two tranches within a three-year structure (Fig. S7). Table S6 highlights a distinction between issuer-default conditions and catastrophe-related payout triggers. For China’s pandemic-related bonds, the conditions described concern issuers’ failure to meet financial obligations. The PEF bonds instead use outbreak-related criteria, including a waiting period, cross-country mortality thresholds, and positive case growth. Natural CAT bonds use industry-loss, indemnity, or parametric triggers tied to specified catastrophe thresholds. These differences in contractual conditions and risk exposure are potentially associated with investors’ pricing of the instruments and provide additional context for the observed premium gap.

Nevertheless, several limitations should be acknowledged. Since 2020, the limited issuance, diversity of pandemic-related instruments, and relatively low secondary-market activity of pandemic bonds have restricted the generalizability of the empirical findings, particularly those related to ESG effects. Limited comparable data on sovereign backing, collateralization, and market segmentation prevent us from identifying their separate contributions to the premium gap between CAT and pandemic bonds. Within the pandemic-bond analysis, issuer-location and currency subsample checks assess sensitivity to sample composition, but small subsamples and limited availability of currency-specific benchmarks prevent us from fully separating liquidity compensation from currency basis, sovereign risk, and local monetary conditions. Furthermore, existing ESG scores often provide limited information in the context of pandemic bonds, as they are not explicitly designed to capture health system capacity or pandemic response effectiveness, which may reduce their observed relevance in terms of pricing. In addition, the health-specific index constructed in this study remains disclosure-based and exploratory in nature and may partly reflect differences in reporting transparency and disclosure incentives rather than purely objective health-system capacity. Finally, classifying market conditions based on the distribution of macroeconomically adjusted price-index residuals may oversimplify the complexity of financial cycles and investor behavior.

## Conclusion

The growth of pandemic bonds demonstrates the potential of financial innovation in pandemic preparedness and response. As climate emergencies and public health crises converge, these instruments may become a complementary component of broader global risk-governance frameworks. However, transferring catastrophe risks to capital markets requires careful management of liquidity-related risks. Using BMA and IML, this study quantifies liquidity premia under substantial model uncertainty and highlights the roles of macroeconomic cyclicality, issuance characteristics, reinsurance-related design features, and sustainability considerations. The results indicate that liquidity premia exhibit pronounced phase-dependent behavior across market cycles, reinforcing the importance of market timing in pandemic bond issuance.

Comparisons with CAT bonds and green bonds reveal persistent gaps in trigger mechanisms, risk modeling, and conventional ESG frameworks. In particular, the limited pricing relevance of aggregate ESG scores in this early-stage market suggests the potential value of more health-oriented sustainability metrics, such as issuer-level health capacity and preparedness indicators. Addressing these structural and informational gaps through improved trigger design, health-aligned sustainability disclosure, and issuance strategies that account for macroeconomic cycles may help enhance the ability of pandemic bonds to transfer public health risks, strengthen health systems, and improve the resilience of global health financing.

## Supplementary Information

Additional file1 (DOCX 1035 kb)

## Data Availability

Data are available upon academic request.

## Abbreviations

BCA: Bias-corrected and accelerated
BMA: Bayesian model averaging
Bps: Basis points
CAT: Catastrophe
CI: Confidence interval
DT: Decision tree
EL: Expected loss
ESG: Environmental, social, and governance
FMI: Fishery Mutual Insurance
GBRT: Gradient boosting regression tree
ICMA: International Capital Market Association
IFFIm: International Finance Facility for Immunization
ILS: Insurance-linked securities
IML: Interpretable machine learning
MAE: Mean absolute error
MLP: Multilayer perceptron
MSE: Mean squared error
OLS: Ordinary least squares
PEF: Pandemic Emergency Financing Facility
PI: Price index
PIP: Posterior inclusion probability
RMSE: Root mean square error
SHAP: SHapley Additive exPlanations
SPV: Special purpose vehicle
S&P: Standard & Poor's
WHO: World Health Organization
XGBoost: Extreme Gradient Boosting
YTM: Yield-to-maturity
