# Supplementary material for: Advancing sustainable financing through pandemic bond: a Bayesian model averaging approach to liquidity premia
Source: Financ Innov. 2026 Nov 6;12(1):167. doi: 10.1186/s40854-026-00991-0 (PMC13743605; doi:10.1186/s40854-026-00991-0)
Supplement: Supplementary file 1 [file 40854_2026_991_MOESM1_ESM.docx]

**Supplementary Materials**

**Advancing sustainable financing through pandemic bonds: a Bayesian model averaging approach to liquidity premia**

**Figure S1.** Disbursements from CCRIF to Caribbean and Central American Countries for addressing natural catastrophes (2017-2023).

**Figure S2.** The issuance mechanism of CAT bonds.

**Figure S3.** The liquidity premia of hurricane bonds and other CAT bonds.

**Figure S4.** The issuer countries and industries of pandemic bonds.

**Figure S5.** Distribution of pandemic bond issuers by health score group

**Figure S6.** The model metrics of ML models.

**Figure S7.** Pandemic bonds summary terms and conditions.

**Table S1.** Literature on pandemic bonds, catastrophe bonds, ESG-linked bonds, and macrofinancial cyclicality.

**Table S2.** Health-specific indicators used to construct the health score.

**Table S3.** Summary of global natural disasters from 2020 to 2023.

**Table S4.** Regression estimates of the best model for pandemic bonds with the highest posterior probabilities.

**Table S5.** Marginal posterior probabilities of individual regressors for wildfire bonds.

**Table S6.** The difference in trigger indemnity between pandemic bonds and natural CAT bonds.

**Supplementary reference:**

Baker M, Bergstresser D, Serafeim G, Wurgler J (2022) The pricing and ownership of U.S. green bonds. J Financ Econ 144(1):14–41. doi:10.1016/j.jfineco.2021.12.002.

Bali TG, Subrahmanyam A, Wen Q (2020) The macroeconomic uncertainty premium in the corporate bond market. J Financ Quant Anal 56(5):1653–1678. doi:10.1017/S0022109020000538.

Berg F, Koelbel JF, Rigobon R (2022) Aggregate confusion: the divergence of ESG ratings. Rev Finance 26(6):1315–1344. doi:10.1093/rof/rfac033.

Braun A (2016) Pricing in the catastrophe bond market: a comparative analysis of yield spread drivers. J Risk Insur 83(3):567–597. doi:10.1111/jori.12088.

Christensen DM, Serafeim G, Sikochi A (2022) Why is corporate virtue in the eye of the beholder? The case of ESG ratings. Account Rev 97(1):147–175. doi:10.2308/TAR-2019-0506.

Döttling R, Kim S (2023) Sustainable finance and bond market liquidity. J Financ Econ 150(2):410–439. doi:10.1016/j.jfineco.2023.02.004.

Ehlers T, Mojon B, Packer F (2022) Green bonds and carbon emissions: exploring the case for a rating system at the firm level. BIS Bulletin No. 49. Bank for International Settlements, Basel. <https://www.bis.org/publ/bisbull49.pdf>. Accessed 22 Mar 2024.

Erikson S (2019) Global health futures? Reckoning with a pandemic bond. Med Anthropol Theory 6(3).

Evans S (2024) Tokio Marine is first Japanese cat bond sponsor to use sustainable development bond. Artemis, 22 March 2024. <https://www.artemis.bm/news/tokio-marine-is-first-japanese-cat-bond-sponsor-to-use-sustainable-development-bond/>. Accessed 22 Mar 2024.

Gilchrist S, Zakrajšek E (2012) Credit spreads and business cycle fluctuations. Am Econ Rev 102(4):1692–1720. doi:10.1257/aer.102.4.1692.

Götze T, Gürtler M (2020) Hard markets, hard times: on the inefficiency of the CAT bond market. J Corp Finance 62:101553. doi:10.1016/j.jcorpfin.2019.101553.

Gürtler M, Hibbeln M, Winkelvos C (2014) The impact of the financial crisis and natural catastrophes on CAT bonds. J Risk Insur 83(3):579–612. doi:10.1111/jori.12057.

Gürtler M, Hibbeln M, Winkelvos C (2016) The impact of catastrophe bonds on reinsurance pricing. J Risk Insur 83(3):641–676. doi:10.1111/jori.12056.

Herrmann M, Hibbeln M (2023) Trading and liquidity in the catastrophe bond market. J Risk Insur 90(2):283–328. doi:10.1111/jori.12407.

Huang S, Tan KS, Zhang J, Zhu W (2024) Epidemic financing facilities: pandemic bonds and endemic swaps. N Am Actuar J 28(3):626–657.

Imran ZA, Ahad M (2023) Safe-haven properties of green bonds for industrial sectors (GICS) in the United States: evidence from COVID-19 pandemic and global financial crisis. Renew Energy 210:408–423. doi:10.1016/j.renene.2023.04.033.

Jeanne O, Sandri D (2023) Global financial cycle and liquidity management. J Int Econ 146:103736.

Zerbib OD (2019) The effect of pro-environmental preferences on bond prices: evidence from green bonds. J Bank Finance 98:39–60. doi:10.1016/j.jbankfin.2018.10.012.

**Figure S1.** Disbursements from CCRIF to Caribbean and Central American Countries for addressing natural catastrophes (2017-2023). Notes: (1) Data are collected from The CCRIF (https://www.ccrif.org/aboutus/ccrif-spc-payouts). (2) Rainfall resulting from tropical cyclones is categorized under the Rainfall Policy. Trough systems are classified separately.

**Figure S2.** The issuance mechanism of CAT bond.

**Figure S3.** The liquidity premia of hurricane bonds, and other CAT bonds.

**Figure S4.** The issuer countries and industries of pandemic bonds.

**Figure S5.** Distribution of pandemic bond issuers by health score group.

**Figure S6.** The model metrics of ML models. Notes: The parameters of the ML models are set as follows: size = 4 and maxit = 500 for ANN; size = 10 and maxit = 1000 for MLP; eta = 0.02, max_depth = 10, gamma = 0.01, and subsample = 0.95 for XGBoost; split = “Gini” for DT; subsample = 0.95, learning_rate = 0.1, max_depth = 10, and nrounds = 200 for GBRT; and learning_rate = 0.1 and nrounds = 200 for LightGBM.

**Figure S7.** Pandemic bonds summary terms and conditions. Notes: The pandemic bonds issued by the World Bank have a total issuance of 320 million with a maturity of three years and matured in July 2020. Structurally, the bonds are divided into two tranches: Tranche A and Tranche B, with 225 million and $95 million issued for each tranche, respectively.

**Table S1.** Literature on pandemic bonds, CAT bonds, ESG-linked bonds, and macrofinancial cyclicality.

| Author (Year) | Focus | Key Findings | Limitations/Research Gap |
| --- | --- | --- | --- |
| Erikson (2019) | World Bank PEF pandemic bond | Unveils the financialization logic and ethical issues of the PEF, criticizing its complexity and opacity | Focuses on governance and ethics, but does not address financial pricing or liquidity risk |
| Huang et al. (2024) | Pandemic bond and endemic swap (COVID-19 & dengue) | Proposes the design of pandemic bonds and endemic swaps to mobilize capital for outbreak response | Emphasizes product design, but lacks analysis of liquidity premium and macrofinancial cycles |
| Evans (2024) | Tokio Marine Holdings’ sustainable CAT bond issuance | Tokio Marine Holdings issued the first CAT bond whose proceeds were invested in sustainable development projects, linking catastrophe risk financing with ESG goals | Shows early ESG integration in CAT bond markets but lacks empirical analysis of liquidity, pricing, or macrofinancial cycles; supports the inclusion of ESG-linked factors in pandemic bond research |
| Döttling & Kim (2023) | Sustainable finance and bond market liquidity | ESG-labeled bonds maintain higher liquidity during crises, indicating that sustainability signals enhance investor confidence under stress | Demonstrates green bond resilience but offers no evidence for pandemic or social bonds |
| Zerbib (2019) | Greenium and liquidity in green bonds | Green bonds exhibit a small but significant liquidity premium (“greenium”) | Evidence limited to environmental assets; social/health bonds remain unexplored |
| Baker et al. (2022) | Ownership and pricing of U.S. green bonds | ESG-oriented investors create persistent greenium and narrower yield spreads | Focuses on U.S. municipal market; does not extend to pandemic or ESG-linked sovereign bonds |
| Berg et al. (2022) | Divergence of ESG ratings | ESG ratings vary widely across data providers, reducing informational reliability | Measurement heterogeneity weakens comparability; no domain-specific ESG framework for health bonds |
| Ehlers et al. (2022) | Carbon intensity and green bond markets | Argues for firm-level ESG rating systems to improve credibility and comparability | Highlights need for standardized disclosure, supporting transparency in pandemic bond design |
| Christensen et al. (2022) | ESG rating subjectivity and rater disagreement | Reveals substantial subjectivity among ESG raters | Reinforces need for domain-specific ESG metrics in health and pandemic financing |
| Götze & Gürtler (2020) | Inefficiency of CAT bond market across hard, neutral, and soft cycles | Finds that CAT bond premia are highly sensitive to market phases: sponsor reputation dominates in hard markets, while financial strength matters in neutral markets; pricing factors lose significance in soft markets | Identifies market-phase heterogeneity but does not link macrofinancial cycles to liquidity premia across bond types; motivates studying pandemic bonds under different market regimes |
| Gilchrist & Zakrajšek (2012) | Examines credit spreads and business cycles in corporate bonds. | Credit spreads widen during recessions via default risk and investor risk aversion channels. | Focused on traditional credit spreads; does not explicitly decompose liquidity premium. |
| Bali et al. (2020) | Studies macroeconomic uncertainty as a pricing factor in corporate bond returns. | Finds a significant “macroeconomic uncertainty premium” in bond cross-sections. | Does not distinguish liquidity effects from uncertainty risk premia. |
| Gürtler et al. (2014) | Investigates impact of financial crises and catastrophes on cat bond risk premia. | Cat bond premia rise with reinsurance cycles and financial crises; correlated with corporate spreads. | Limited liquidity analysis; focuses on cyclical risk correlation. |
| Imran & Ahad (2023) | Studies safe-haven behavior of green bonds during crises. | Green bonds hedge systemic shocks and combine environmental and stabilizing functions. | Concentrates on hedging properties rather than liquidity channels. |
| Jeanne & Sandri (2023) | Global financial cycle and liquidity management in emerging markets | Shows that emerging markets can buffer global liquidity shocks by accumulating foreign reserves in booms and selling them in busts; optimal policy expands gross flows to stabilize asset prices | Focuses on macropolicy liquidity management, not bond-level liquidity premia; offers macro rationale for including global-cycle heterogeneity in bond-pricing models |
| Herrmann & Hibbeln (2023) | Bid-Ask Spread (TRACE data) used as a direct market-based measure of liquidity. | First paper to quantify cat bond liquidity premium empirically using real trade data. | Requires active secondary trading data; not applicable to thinly traded or pandemic bonds. |
| Braun (2016) | YTM-LIBOR differential as an indirect proxy capturing both liquidity and model risk. | Highlights liquidity as part of the residual spread beyond expected loss; robust for illiquid markets. | Cannot isolate pure liquidity component; depends on modeled default risk accuracy. |
| Gürtler et al. (2016) | Issue volume & maturity used as structural proxies for liquidity. | Larger issue size and shorter maturity linked to lower spreads, suggesting better liquidity. | Indirect measure; lacks transaction data, limiting precision. |

**Table S2.** Health-specific indicators used to construct the health score.

| Indicator Code | Indicator Name | Definition |
| --- | --- | --- |
| H1 | Health system infrastructure capacity | Whether the issuer discloses commitments or investments in strengthening health system infrastructure, including healthcare facilities and essential operational conditions. |
| H2 | Health workforce capacity and protection | Whether the issuer reports policies or investments related to health workforce capacity, training, protection, or emergency mobilization of health personnel. |
| H3 | Disease surveillance and health information systems | Whether the issuer discloses the existence or support of disease surveillance systems, health information systems, or public health data and early-warning mechanisms. |
| H4 | Public health emergency preparedness and coordination | Whether the issuer reports institutional arrangements or governance mechanisms for public health emergency preparedness, response coordination, or crisis management. |
| H5 | Health-related legal and regulatory capacity | Whether the issuer discloses health-related regulatory or legal frameworks, including infection prevention, international health regulations, or medicine and health product regulation. |
| H6 | Public financing commitment to health | Whether the issuer reports explicit public financing commitments, budgetary allocations, or fiscal support for health or public health systems. |
| H7 | Access to essential health services and financial protection | Whether the issuer references policies or objectives aimed at improving access to essential health services or reducing financial barriers to healthcare (e.g. UHC). |
| H8 | Health-aligned use of proceeds | Whether bond proceeds are explicitly allocated to health-related or healthcare-related projects, activities, or systems. |
| H9 | Health-related project selection and evaluation process | Whether the issuer explains how projects are selected or evaluated with respect to health objectives or target health-related populations. |
| H10 | Health-related allocation or impact reporting | Whether the issuer provides allocation or impact reporting related to health or healthcare projects financed by bonds. |

**Table S3**. Summary of global natural disasters from 2020 to 2023.

| Year | Frequency | Affected persons (million) | Deaths | Direct economic loss (USD bn) |
| --- | --- | --- | --- | --- |
| 2020 | 313 | 98.97 | 15082 | 173.13 |
| 2021 | 367 | 104.17 | 10492 | 252.14 |
| 2022 | 321 | 185.96 | 30759 | 223.84 |
| 2023 | 399 | 93.10 | 86473 | 202.70 |

**Table S4.** Regression estimates of the best model for pandemic bonds with the highest posterior probabilities.

| Variable | Pandemic | Soft | Neutral | Hard |
| --- | --- | --- | --- | --- |
| Intercept |  |  |  | 12.07**^.^** |
| Volume |  | 0.12** |  | -0.45 |
| Maturity | 0.13*** | 0.05**^.^** | 0.12* | 0.008 |
| Coupon |  | 0.61*** |  |  |
| Type | 2.37** | -0.80 | 2.70**^.^** |  |
| Issue market |  | 1.66** |  |  |
| R^2^ | 0.12 | 0.90 | 0.09 | 0.10 |
| Prob | 0.29 | 0.01 | 0.10 | 0.24 |

Notes: ***, **, * and ^.^ indicate significance at the 0.1%, 1%, 5% and 10% levels.

**Table S5**. Marginal posterior probabilities of individual regressors for wildfire bonds.

| Ranking | Variable | Effect | Prob (%) |
| --- | --- | --- | --- |
| 1 | Coupon | 0.77 | 99.99 |
| 2 | Asia/Australia | 21.54 | 99.99 |
| 3 | EL | 0.56 | 92.07 |
| 4 | Volume | -1.28 | 63.35 |
| 5 | Rating 2 | 0.88 | 32.07 |
| 6 | Maturity | -0.21 | 30.83 |
| 7 | S&P 500 | -0.005 | 30.04 |
| 8 | Term | 0.08 | 22.80 |
| 9 | Corp | -0.37 | 20.71 |
| 10 | North America | 0.32 | 20.08 |
| 11 | Rating 3 | 0.52 | 20.06 |
| 12 | Rating 1 | -0.13 | 18.38 |
| 13 | Europe | 0.13 | 18.36 |

**Table S6**. The difference in trigger indemnity between pandemic bonds and natural CAT bonds.

| Bond | Trigger Indemnity |
| --- | --- |
| Pandemic Bonds in China | Issuer or its subsidiaries fail to meet their financial obligations, such as timely repayment of debts. |
| World Bank pandemic Bond catastrophe bond | 1) 12 weeks after start of event. 2) At least two countries affected by at least 20 deaths each. 3) Case growth rate above zero. |
| CAT Bonds | 1) Assessed total industry loss exceeds the predetermined threshold.  2) The insurance company's actual losses exceed the predetermined threshold.  3) A named disaster impacts specified areas and the predefined parameters (such as wind speed or earthquake magnitude) exceed certain thresholds. |

Notes: Data are collected from Artemis Deal Directory and Wind.
